# Biofilm forming rhizobacteria affect the physiological and biochemical responses of wheat to drought

**DOI:** 10.1186/s13568-022-01432-8

**Published:** 2022-07-14

**Authors:** Esmaeil Karimi, Nasser Aliasgharzad, Ezatollah Esfandiari, Mohammad Bagher Hassanpouraghdam, Thomas R. Neu, François Buscot, Thomas Reitz, Claudia Breitkreuz, Mika T. Tarkka

**Affiliations:** 1 Department of Soil Science, University of Maragheh, Maragheh, Iran; 2 Department of Soil Science, University of Tabriz, Tabriz, Iran; 3 Department of Agronomy and Plant Breeding, University of Maragheh, Maragheh, Iran; 4 Department of Horticultural Sciences, University of Maragheh, Maragheh, Iran; 5 Department of River Ecology, Helmholtz Centre for Environmental Research-UFZ, Magdeburg, Germany; 6 Department of Soil Ecology, Helmholtz Centre for Environmental Research-UFZ, Halle, Germany; 7 German Centre for Integrative Biodiversity Research (iDiv) Halle-Jena-Leipzig, Leipzig, Germany

**Keywords:** Biofilm forming PGPR, Wheat, Drought response, Antioxidant enzymes, Harvest index, Root tissue density

## Abstract

Plant growth promoting rhizobacteria (PGPR) can attenuate the adverse effects of water deficit on plant growth. Since drought stress tolerance of bacteria has earlier been associated to biofilm formation, we aimed to investigate the role of bacterial biofilm formation in their PGPR activity upon drought stress. To this end, a biofilm-forming bacterial collection was isolated from the rhizospheres of native arid grassland plants, and characterized by their drought tolerance and evaluated on their plant growth promoting properties. Most bacterial strains formed biofilm in vitro. Most isolates were drought tolerant, produced auxins, showed 1-aminocyclopropane-1-carboxylic acid (ACC) deaminase activity and solubilized mineral phosphate and potassium, but at considerably different levels. Greenhouse experiments with the most promising isolates, B1, B2 and B3, under three levels of water deficit and two wheat varieties led to an increased relative water content and increased harvest index at both moderate and severe water deficit. However, the bacteria did not affect these plant parameters upon regular watering. In addition, decreased hydrogen peroxide levels and increased glutathione S-transferase activity occurred under water deficit. Based on these results, we conclude that by improving root traits and antioxidant defensive system of wheat, arid grassland rhizospheric biofilm forming bacilli may promote plant growth under water scarcity.

**Supplementary Information:**

The online version contains supplementary material available at 10.1186/s13568-022-01432-8.

## Key points

- Numerous biofilm forming PGPR reside in grass rhizospheres from arid grasslands.
- Drought tolerance of wheat is enhanced by bacterial inoculations.
- Wheat variety and the level of drought stress modify the plant’s response to the bacteria.

**Supplementary Information:**

The online version contains supplementary material available at 10.1186/s13568-022-01432-8.

## Introduction

Water deficit is expected to cause serious problems for crops on more than 50% of the earth’s arable lands by 2050 (Vinocur and Altman 2005). With ongoing global climate change the severity, frequency and duration of drought periods are predicted to further increase (Vaghefi et al. 2019). Based on the national climate change project, Iran will face an average increase of 2.6% in annual temperatures and 35% decline in precipitation by 2050 (NCCOI), making crop production without irrigation even more challenging in the future. Thus, understanding and improving plant survival as well as growth under restricted water availability is of central significance in contemporary plant science and consequently is a prerequisite for efficient plant production (Mancosu et al. 2015).

Plant growth promoting rhizobacteria (PGPR) facilitate plant growth through growth regulation, biofertilization and biocontrol (Glick 2014). Some PGPR protect plants from environmental stress, including water deficit (Budak et al. 2013; Cooper et al. 2014). The need for microbial-based approaches to mitigate drought stress has led to increased attention for enhancing crop productivity and providing stress resistance by PGPR (Ngumbi and Kloepper 2016; Kaushal and Wani 2016; de Vries et al. 2020). The term “Induced Systemic Tolerance” has been attributed for the changes induced by microorganisms in plants that are associated with enhanced plant tolerance to drought stress (Vacheron et al. 2013; Vurukonda et al. 2016; Dimkpa et al. 2009). The underlying mechanisms include modulation of root system architecture by the production of auxins (Postma and Lynch 2011) and ACC-deaminase (Glick 2014), differential expression levels of stress-responsive genes (Shinozaki and Yamaguchi-Shinozaki 2007), production of rhizobacterial exopolysaccharides (EPS), biofilm formation (Vardharajula et al. 2009), as well as osmotic adjustment of plant roots (Shintu and Jayaram 2015).

Biofilms are microbial aggregates usually associated with an interface and consist of single or multiple species (Marshall 1976). In general, the microbial cells adhere to biotic or abiotic surfaces. Upon intimate contact with each other and quorum sensing, a matrix of extracellular polymeric substances (EPS) will be formed around the consortium (Flemming and Wingender 2010). The formation of EPS leads to physiological changes in microbial cells and enhanced stress tolerance of the cells (Schnider-Keel et al. 2001). Kasim et al. (2016) reported that formation of biofilm under varying stress conditions is a significant strategy adopted by bacterial strains for their successful survival in plant rhizosphere. Biofilm forming capacity is frequent among the bacteria with mucous colonies and the colonization pattern of plant surfaces by plant-associated microbial populations shows similarities to the formation of biofilms on abiotic surfaces (Molina et al. 2003). The extracellular matrix of bacteria in biofilms contains carbohydrates, oligo and polysaccharides that may play various roles in bacteria–plant interactions such as improving water availability in the rhizosphere (Timmusk et al. 2014). As a consequence, there is a great potential in isolating rhizosphere biofilm forming PGPR from unexplored, drought affected environments.

The principal idea of this project was to isolate bacteria that promote plant growth under drought stress, support plant nutrition by solubilizing nutrients, promoting plant growth and form biofilms. In order to obtain a diverse collection of root-associated and drought-tolerant bacteria, the bacteria were isolated from roots of five grass species (*Gramineae*) grown in natural grassland systems on low moisture soils of East Azerbaijan, Iran. Subsequently, the growth promoting abilities of the isolated strains were tested on two wheat cultivars in a pot experiment. We expected that PGPR in biofilm mode should perform well under water deficit, and that the plant growth-dependence of these bacteria is negligible under normal watering.

## Materials and methods

### Isolation of biofilm forming bacteria from rhizosphere soil

The samples were collected from East Azerbaijan province of Iran (Additional file 1: Fig. S1) in late July 2015. The climate of this region is characterized by low precipitation and high temperature in summer (Vaghefi et al. 2019). The four grasslands selected for this study were unmanaged. In total, 50 rhizosphere soil samples of actively growing *Gramineae* plants were harvested in the following way. After the excavation of the root system, four lateral roots from the top soil layer (1–7 cm from soil surface) were shaken to remove most of the attached soil. The first 10 mm of these four lateral roots were cut into 10 mL physiological solution (0.85% NaCl) and shaken on rotary shaker at 120 rpm for 10 min. Ten microliter of this solution was plated on sterile trypticase soy agar (TSA) and incubated for 3 days at 30 °C. To enrich potentially biofilm forming bacteria only mucous colonies were picked, streaked on sterile TSA plates and purified.

### Biofilm formation assay

Biofilm formation was distinguished in two forms, bacteria forming pellicles and those that are adherent to the surface. All isolates were cultivated aerobically overnight in TSB. The bacterial cultures were diluted with fresh TSB medium to obtain an optical density of 0.5 at 600 nm, and a bacterial suspension (100 µL) was inoculated in three replicates per strain into polystyrene 96-well microtiter plates. The plates were incubated at 30 °C for 18 h. Isolates that were able to form pellicle were recorded as “robust biofilm forming”. Adherent bacteria were grouped in four categories by crystal violet staining according to O'Toole (2011) at 550 nm wavelength, based on OD (optical density) and ODC (optical density cutoff, i.e. the value of the assay without bacteria). The categories were non-adherent: OD ≤ ODC, weakly adherent: ODC < OD ≤ (2 × ODC); moderately adherent: (2 × ODC) < OD ≤ (4 × ODC), and strongly adherent: (4 × ODC) < OD (Basson et al. 2008).

### Screening for drought tolerance

By the addition of sorbitol or polyethylene glycol (PEG; molecular weight 8000; Sigma, St Louis, MO) to the TSA growth medium, water potential was lowered. Sorbitol addition showed if the strain morphology changed upon drought, with an assay according to Kavamura et al. (2013). PEG addition was used to measure the growth rates of different strains under low water potential, which was reached by addition of 0.5% PEG solution (PEG8000, Promega, WI, USA).

### Root growth and plant nutrition related activities

Indole acetic acid (IAA) production was estimated following the protocol described by Brick et al. (1991). Twelve strains per plate were plated onto 10% TSB amended with 5 mM of l-tryptophan (Merck, Germany), overlaid with a nitrocellulose membrane (0.45 μm pore size, Sartorius) and incubated at 28 °C for 36 h. After bacterial growth had occurred, the membrane was removed from the plate and treated with Salkowski reagent (2% (w/v) 0.5 M FeCl_3_ in 35% perchloric acid) for 30 min at room temperature. Bacteria producing IAA were identified by the presence of a red halo on the membrane corresponding to the position of the IAA-producing colony. Ability of isolates to the production of ACC deaminase were examined according to the method of Chen et al. (2008) in MSA medium with ACC (0.5 g L^−1^) (ACC+) and that without ACC (ACC−). Siderophore release was determined as described by Schwyn and Neilands (1987). The mineral P-solubilizing ability of the strains was determined in Pikovskaya’s liquid medium amended with 0.5% [Ca_3_(PO_4_)_2_] as described by Mehta and Nautiyal (2001). Quantitative estimations of solubilized K in Aleksandrov liquid medium occurred according to Parmar and Sindhu (2013).

### Greenhouse experiment

A greenhouse experiment was conducted to evaluate the impact of three most promising bacterial isolates on wheat under controlled conditions. Wheat was selected for the greenhouse study, since it is the major crop cultivated in Iran, accounting for over 60% of cereal production (FAO 2020). Nutrient rich sandy loam soil (Additional file 1: Table S1) was collected from the research field station of the University Maragheh (37.3787° N, 46.2756° E). The experiment was conducted in a full-factorial, randomized block design. The factors included biofilm forming bacterial isolates with a non-inoculated control and three bacterial isolates: *Peribacillus simplex* strain 16-1 with the German Collection of Microorganisms and Cell Cultures accession DSM113966 and the abbreviation of B1 in the greenhouse experiments, *Bacillus pumilus* 38-2 DSM113967 and B2, and *Peribacillus simplex* 54-1 DSM113968 and B3. Partial 16S rDNA sequences of the isolates, determined according to Breitkreuz et al. (2020) were used for naming the isolates and are given in Additional file 1: Table S2. Furthermore, two cultivars of wheat (*Triticum aestivum* L.) cvs. Kohdasht (C1) and Chamran (C2). Chamran and Kohdasht representing spring wheat cultivars that are frequently cultivated in Iran in regions with a mild winter. They showed a comparable tolerance to water deficit in a greenhouse assay (Johari-Pireivatlou and Maralian 2011), but differ in areas of cultivation. Whereas Chamran cv. is suitable for planting in hot and humid regions, Kohdasht cv. is more frequently used for planting in hot and dry regions. Furthermore, three soil moisture levels of 80%, 50%, and 30% of soil available water content (AWC), termed as normal, moderate and low watering were used. AWC was calculated by differences between field capacity water and permanent wilting point water of the soil. These two soil water indexes were measured by a pressure plate. Soil water contents of the pots were adjusted by weighing them.

Bacteria were cultivated in TSB at 28 °C with vigorous shaking at 280 rpm for 24 h. Bacterial cells were pelleted by centrifugation, washed once with 0.85% NaCl, and resuspended in a sterile 0.85% NaCl solution to the population density of 5 × 10^8^ colony forming units per milliliter (CFU mL^−1^). After surface sterilization of wheat seeds with 1% NaClO for 10 min and 45 s in 70% ethanol, they were incubated in the 5 × 10^8^ colony forming units CFU mL^−1^ bacterial suspension for 5 min. Then, the seeds were sown into plastic pots filled with 5 kg autoclaved sandy loam soil. Water stress treatment was started at the beginning of headlines Biologische Bundesanstalt, Bundessortenamt und Chemische Industrie (BBCH scale 51), and continued up to the end of growth. Watering treatment was carried out based on pot weight and the targeted soil available water content.

### Plant harvest and measured physiological and biochemical parameters

After 12 days of water deficit treatment, young leaves were harvested and their relative water content (RWC) was determined by the difference between fresh and leave turgor weight according to Marchiol et al. (1996). Membrane stability index was determined from fresh leaves by recording the electrical conductivity differences among leaf ions leaching in double distilled water at 40 °C and 100 °C water after 15 min (Devi and Kar 2013). For other parameters that are explained below, young leaves were frozen in liquid N_2_ and stored at − 20 °C before analysis. Degree of lipid peroxidation was estimated by evaluating the amount of malondialdehyde, determined by a colorimetric method. For this, 0.5 g of grounded leaf samples were homogenized in 5 ml of distilled water and an equal volume of 0.5% thiobarbituric acid in 20% trichloro acetic acid (TCA) was added. The solution was incubated at 95 °C for 30 min. Subsequently, the adsorption was measured at 532 nm (Stewart and Bewley 1980). Hydrogen peroxide levels were determined in homogenized leaf extract in ice bath with 5 ml 0.1% (w/v) TCA and its reaction with potassium iodide according to Sergiev et al. (1997). For superoxide dismutase (SOD), catalase (CAT) and glutathione peroxidase (GPX) extraction, leaf samples (0.5 g) were homogenized in ice-cold 0.1 M phosphate buffer (pH 7.5) containing 0.5 mM Ethylenediaminetetraacetic acid (EDTA) with pre-chilled pestle and mortar. Each homogenate was transferred to centrifuge tubes and was centrifuged at 4 °C for 15 min at 15,000*g*. The supernatant was used for various enzyme activity assays. SOD activity was determined according to Sen Gupta et al. (1993) by measuring the inhibition of NBT (nitro blue tetrazolium) reduction at 560 nm. One enzyme unit was defined as the amount of enzyme which could cause 50% inhibition of the photochemical reaction. Catalase (CAT) and peroxidase (POX) activities were assayed as described by Aebi (1984) and Panda et al. (2003), respectively. The method of Yoshimura et al. (2000) and Carmagnol et al. (1983) were employed to assay ascorbate peroxidase (APX) and glutathione S-transferase (GST), respectively. The concentration of protein was determined by the method of Bradford (1976) using bovine serum albumin (BSA) as a standard.

### Wheat growth and seed production

At the end of the experiment, grain and straw biomasses were measured by its weight. The Harvest index (HI) was calculated by the following formula:

$$\mathrm{HI}=\frac{\mathrm{Ye}}{\mathrm{Yb}}\times 100$$

where is, HI = Harvest index, Ye = Economical yield (grain biomass) and Yb = biological yield (total biomass, i.e. straw + grain).

The root systems of each pot were gently washed with tap water, and the root volume was measured by a graduated cylinder in the water by variation of water volume with root and without root. Fresh root weight and oven-dried weight were measured for each pot, root tissue density and root water content percentage were calculated by these relations:

$$\mathrm{Root\, tissue\, density }\left({\mathrm{g cm}}^{-3}\right)=\frac{\mathrm{root \,dry\, weight }\left(\mathrm{g}\right)}{\mathrm{fresh\, root\, volume }\left({\mathrm{cm}}^{-3}\right)}$$

$$\mathrm{Root \,water\, content }(\mathrm{\%})=\frac{\mathrm{root\, fresh\, weight}-\mathrm{root\,dry \,weight}}{\mathrm{root \,dry\, weight}}\times 100$$

### Statistics

MSTAT software version 6.5.1 (University of Wisconsin Medical School, Madison, Wisconsin) was used to analyse the data obtained from each section and Excel software was used to draw the graphs. The means were compared using Duncan’s test at 5% probability level.

### Confocal laser scanning microscopy

For imaging the root samples in hydrated state, a TCS SP5X confocal laser scanning microscope (Leica, Germany) was available. The system was equipped with an upright microscope and a super continuum light source. The device was controlled via the LAS AF 2.4.1 software (Leica Microsystems, Wetzlar, Germany). Datasets were recorded by using 25 × NA 0.95 and 63 × NA 1.2 water immersion lenses. Excitation was at 483 nm, and emission signals were collected from 478–488 nm (reflection) and 495–550 nm (SybrGreen). Root samples were stained and mounted in a 2 mm deep cover well chamber. Optical sections were recorded at 0.5 µm or 1 µm interval. Projections were made by using the software Imaris ver. 9.1.2 (Bitplane, Switzerland).

## Results

### Isolation of beneficial bacterial strains

A comparable number of rhizosphere bacteria was isolated from the roots of five *Gramineae* plants, *Lolium temulentum, Avena fatua, Bromus tectorum, Hordeum murinum* and *Agropyron caninum*, and in total 130 of them represented mucoid bacterial strains, forming moist sticky colonies. Out of the 130 mucoid isolates, 24 strains (18.5%) formed solid surface-associated (SSA) biofilms, and 40 strains (30.8%) formed pellicles on the surface of the growth medium. SSA biofilms develop on biotic and abiotic solid surfaces (Stewart and Franklin 2008), and when grown in a liquid under aerobic and static conditions bacteria may form a SSA biofilm or sediment at the bottom of the container (Additional file 1: Figs. S2 and S3). By contrast, some bacteria exploit the air–liquid interface to build floating biofilms, which are termed pellicles (Armitano et al. 2014). The 64 SSA biofilm and pellicle forming isolates were analyzed further.

### Resistance against low water potential

The 64 isolates showed potential adaptation to an environmental stress typical of arid grassland soils, i.e. a tolerance against high osmotic potential. The 64 isolates grew on polyethylene glycol supplemented medium, but only 37 of them (57.8%) proliferated on a medium supplemented with 30% (w/v) sorbitol (Additional file 1: Table S3). The isolates showed differences in their colony morphology in different sorbitol concentrations (Additional file 1: Fig. S4). Colony diameter of pellicle forming bacteria increased at higher sorbitol concentrations, with looser and slimier colonies, indicative of exopolysaccharide (EPS) production (Additional file 1: Fig. S4).

### Plant growth promoting activities of the isolates

All of the 64 osmotic stress tolerant isolates produced auxin indole-3-acetic acid (Table 1). Most (n = 62) of them showed 1-aminocyclopropane-1-carboxylate (ACC) deaminase activity (Additional file 1: Table S4). In line with our study almost all isolates (n = 62) solubilized phosphorus from Ca_3_PO_4_, and all 64 isolates released the K from muscovite mineral (Additional file 1: Table S4).

**Table 1:** PGP abilities of the biofilm forming isolates (64 isolates)

PGP abilities of 64 biofilm forming bacteria	Quantities
Auxin production ability (in the presence of l-tryptophan)	All 64 isolates (6–65 µg mL^−1^ during 36 h)
ACC deamination rate	62 isolates (17.34–275.4 µg L^−1^ α-ketobutyrate during 36 h)
Solubilization of P from Ca_3_(PO_4_)_2_	62 isolates (1.2–251 mg L^−1^ during 72 h)
Solubilization of K from muscovite	All 64 isolates (90 to 200 mg L^−1^ during 7 days)

### Inoculation of beneficial strains and their effects on wheat performance under drought

Next we tested whether the grass rhizosphere bacteria promoted wheat drought tolerance in a greenhouse experiment with three watering levels and two wheat varieties. Three biofilm producing bacterial isolates were selected for the wheat inoculation experiment: (i) *P. simplex* DSM 113,966 strain 16-2 (B1), characterized by strong biofilm forming activity, high auxin production and muscovite solubilization, (ii) related to the *B. pumilus* group DSM 113,967 strain 38-2 (B2) with high muscovite and mineral P solubilization, and strong auxin production, and (iii) related to *Peribacillus simplex* DSM 113,968 strain 54-1 (B3) with high muscovite and strong auxin production levels (Additional file 1: Table S4). These strains had high IAA production and ACC deaminase activities, as well as high P and K solubilization levels.

### Bacterial isolates alter wheat physiological and biochemical traits

According to an analysis of variance (Table 2), watering affected relative water content (RWC), membrane stability index (MSI), malondialdehyde (MDA) concentration, hydrogen peroxide formation, and enzyme activities in wheat leaves. The latter included ascorbate peroxidase (APX), guaiacol peroxidase (GPX) and glutathione-S-transferase (GST). Bacterial inoculation affected RWC, MSI, MDA concentration, and enzyme activities in wheat leaves, with effects on superoxide dismutase (SOD), catalase (CAT), GPX and GST activities. Wheat variety defined MSI, MDA level, hydrogen peroxide formation, and enzyme activities in wheat leaves, with effects on SOD, CAT, APX, GPX and GST activities. Furthermore, there was a strong interaction between the treatments of watering, bacterial inoculation and wheat variety (Table 2).

**Table 2:** Analysis of variance (ANOVA) for the effects of treatments on physiological characteristics of wheat

Source of variation	df	Mean of square								
		RWC	MSI	MDA	H_2_O_2_	SOD	CAT	APX	GPX	GST
Watering	2	9556**	7449**	105,568**	3725**	70^ns^	2^ns^	175**	90**	74,379**
Bacteria	3	559**	381**	5682**	6^ns^	1883**	140**	7^ns^	121**	61,139**
Wheat cv	1	55^ns^	5151**	24,793**	379**	350**	25**	3037**	191**	462,544**
Watering × Bacteria	6	69^ns^	37^ns^	1463**	56**	317**	22**	10^ns^	17^ns^	49,301**
Wheat cv. × Watering	2	441**	1738**	13,546**	279**	63^ns^	8**	467**	6^ns^	8557**
Bacteria × Wheat cv	3	139**	25^ns^	272**	39*	2102**	8*	10^ns^	20^ns^	43,885**
Bacteria × Wheat cv. × Watering	6	30^ns^	130**	221**	12^ns^	369**	13*	26^ns^	14^ns^	23,515**
^+^CV (%)		9.3	6.3	2.6	17.8	11.9	21.6	23	18.1	4.8

*RWC* relative water content, *MSI* membrane stability index, *MDA* malondialdehyde, *H*_*2*_*O*_*2*_ hydrogen peroxide, *SOD* superoxide dismutase, *CAT* catalase, *APX* ascorbate peroxidase, *GPX* guaiacol peroxidase, *GST* glutathione-S-transferase, *CV* cofficient of variation

Asterisks indicate significant differences according to ANOVA at the levels of p < 0.05* and p < 0.01**

### Relative water content (RWC)

**Fig. 1:**
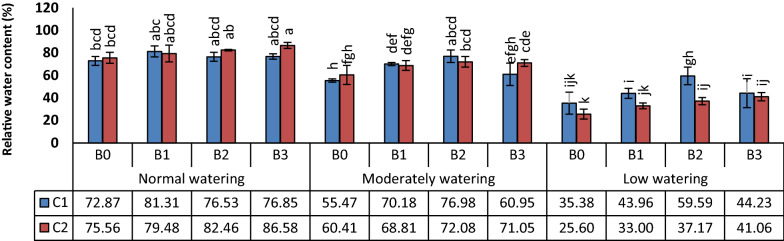
Effect of biofilm forming isolates on relative water content of wheat leaves (RWC). Wheat seedlings of the varieties Kohdasht (C1) and Chamran (C2) were inoculated with the rhizobacteria strain 16-1 (B1), strain 38-2 (B2) and, strain 54-1 (B3) or without inoculation (B0). Different letters indicate a significant difference according to ANOVA and Duncan’s test (p < 0.05)

A significant increase in wheat RWC was observed in all bacterial treatments over the control. However, their effects on this plant trait were affected by the wheat cultivars. After bacterial inoculation, mean RWC value increased by 20% and 17.9%, respectively, for Kohdasht (C1) and Chamran (C2) varieties compared to the respective non-inoculated control (Fig. 1).

### Membrane stability index and malondialdehyde content

**Fig. 2:**
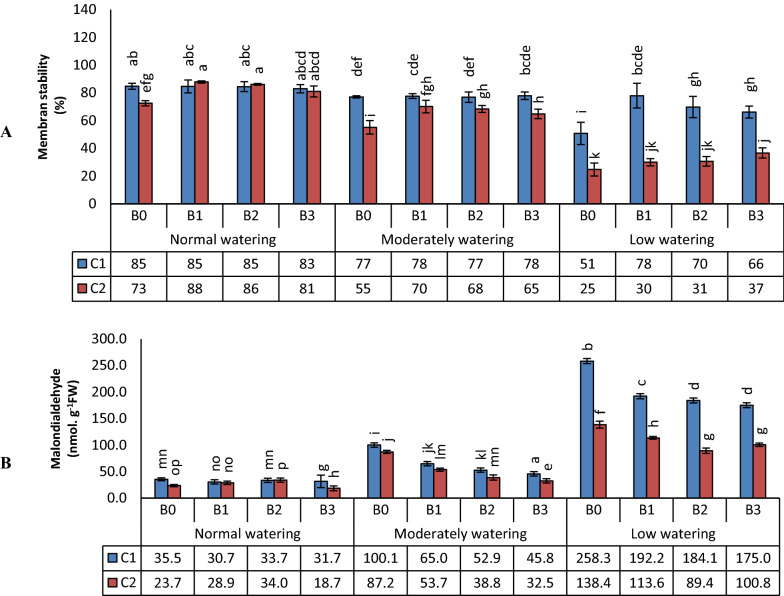
The effect of biofilm forming isolates on wheat membrane stability (**a**) and malondialhyde production (**b**). Wheat seedlings of the varieties Kohdasht (C1) and Chamran (C2) were grown at three levels of water availability, either without (B0) or with the rhizobacteria strain 16-1 (B1), strain 38-2 (B2) and, strain 54-1 (B3). Different letters indicate a difference according to ANOVA and Duncan’s test (p < 0.05)

Water deficit decreased MSI but increased MDA, which are both markers for membrane damage (Miller et al. 2010). The three bacteria enhanced the MSI index in water deficit condition for cultivar 1, but only B3 for wheat cultivar 2 (Fig. 2a). Malondialdehyde contents decreased by the treatments with the three bacteria in both wheat cultivars (Fig. 2b).

### H_2_O_2_ concentration and antioxidant enzymatic activity

**Fig. 3:**
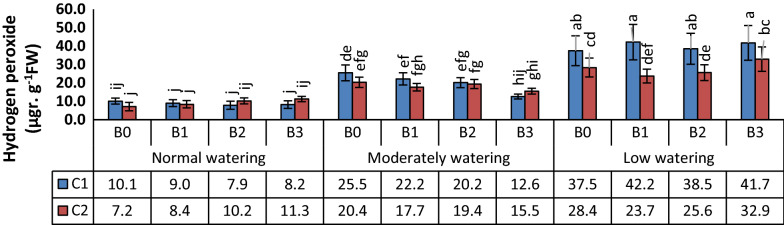
Effects of watering, wheat cultivar and three biofilm forming *Bacillus* isolates on H_2_O_2_ concentration. Wheat seedlings of the varieties Kohdasht (C1) and Chamran (C2) were grown at three levels of water availability and without (B0) or with the rhizobacteria strain 16-1 (B1), strain 38-2 (B2) and, strain 54-1 (B3). Different letters indicate a difference according to ANOVA and Duncan’s test (p < 0.05)

H_2_O_2_ concentration increased in the leaves of wheat upon drought treatments (Fig. 3). The effect of bacteria was low. In moderate water deficit, treatment with the isolate B3 led to a decrease in the concentration of H_2_O_2_ in wheat leaves, specifically in cv. Kohdasht (C1) (Fig. 3).

**Fig. 4:**
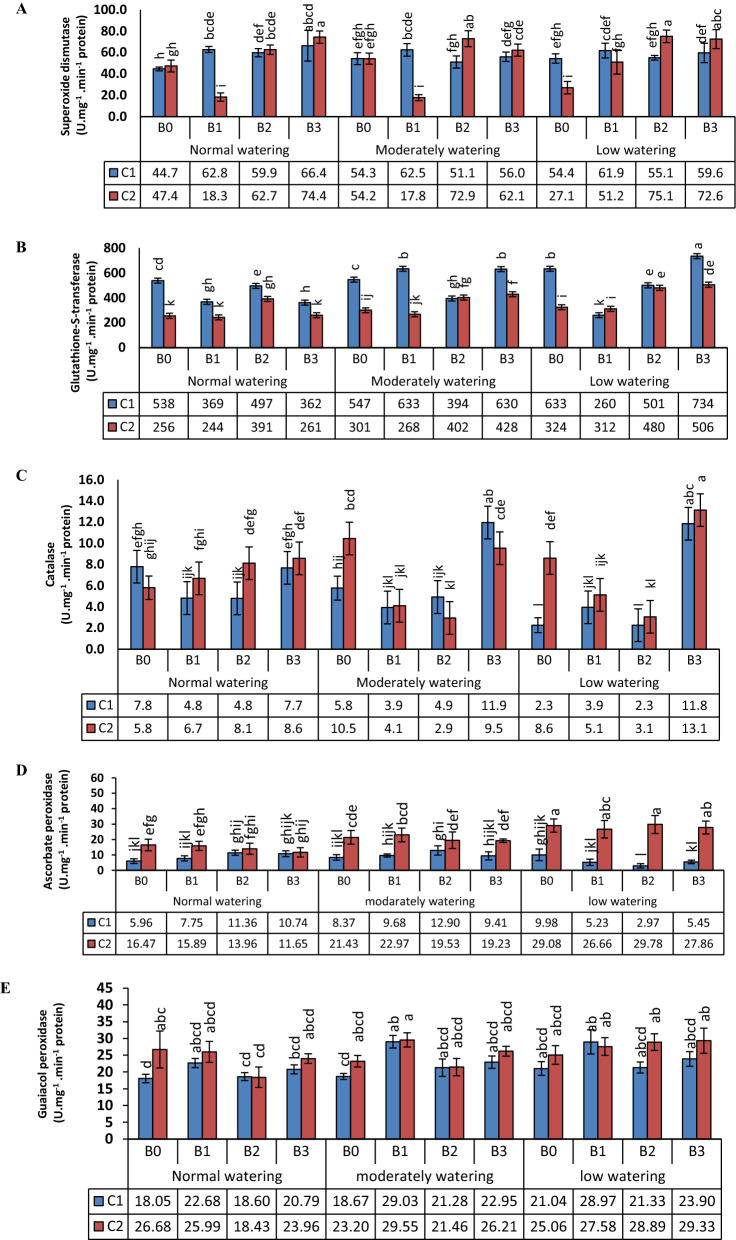
Superoxide dismutase (**a**), glutathione-S-transferase (**b**), catalase (**c**), ascorbate peroxidase (**d**) and guaiacol peroxidase (**e**) activities. Wheat seedlings of the varieties Kohdasht (C1) and Chamran (C2) were grown at three levels of water availability and without (B0) or with the rhizobacteria strain 16-1 (B1), strain 38-2 (B2) and, strain 54-1 (B3). Different letters indicate a difference according to ANOVA and Duncan’s test (p < 0.05)

Superoxide dismutase (SOD) activity (Fig. 4a) under normal watering increased for both varieties with inoculated bacteria compared to non-inoculated ones. An exception was found for bacteria B1 in Chamran (C2) with decreased SOD compared to the control. The same pattern was found for moderate water availability (Fig. 4a). Glutathione-S-transferase (GST) activity (Fig. 4b) increased by drought (low watering) in C1 as well as for moderately and low watering in C2 if compared with non-inoculated wheat in normal watering. The activity levels were in general lower for C2 than C1. With normal watering, the GST activity was suppressed by all bacteria for C1, but increased by B2 for C2. With moderately watering, the GST activity was suppressed by B1 and B3 and enhanced by B2 for C1, but increased by B2 and B3 for C2. Under low watering, the GST activity was suppressed by B1 and B2 and enhanced by B3 for C1, and increased by B2 and B3 for C2. Catalase (CAT) activity (Fig. 4c) increased by drought (low watering) in C1, but decreased by drought (moderately and low watering) in C2 compared to non-inoculated wheat in normal watering. With normal watering, the CAT activity was suppressed by B1 and B2 for C1, but increased by B3 for C2. Under moderate and low watering conditions, the CAT activity was increased by B3 for C1 and partly C2, but decreased by B1 and B2 for C2 (Fig. 4c). The interactions between the treatments were neither significant for GPX nor APX. In all watering regimes these enzyme activities were higher for the cultivar C2 (Fig. 4d and e).

### Root growth and yield of wheat

**Fig. 5:**
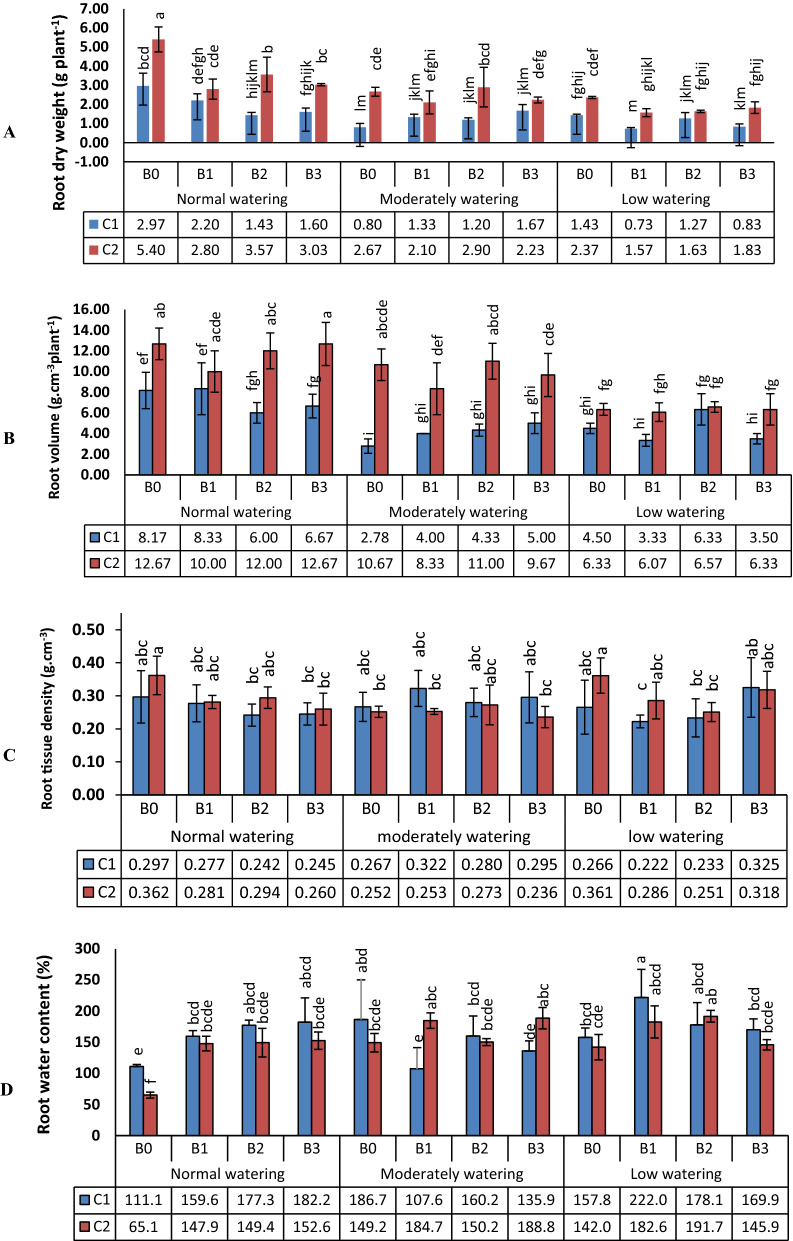
Effects of three biofilm forming rhizosphere bacteria on root dry weight of wheat (**a**), root volume (**b**), root tissue density (**c**) and root water content (**d**). Wheat seedlings of the varieties Kohdasht (C1) and Chamran (C2) were grown at three levels of water availability and without (B0) or with the rhizobacteria strain 16-1 (B1), strain 38-2 (B2) and, strain 54-1 (B3). Different letters indicate a significant difference according to ANOVA and Duncan’s test (p < 0.05)

**Fig. 6:**
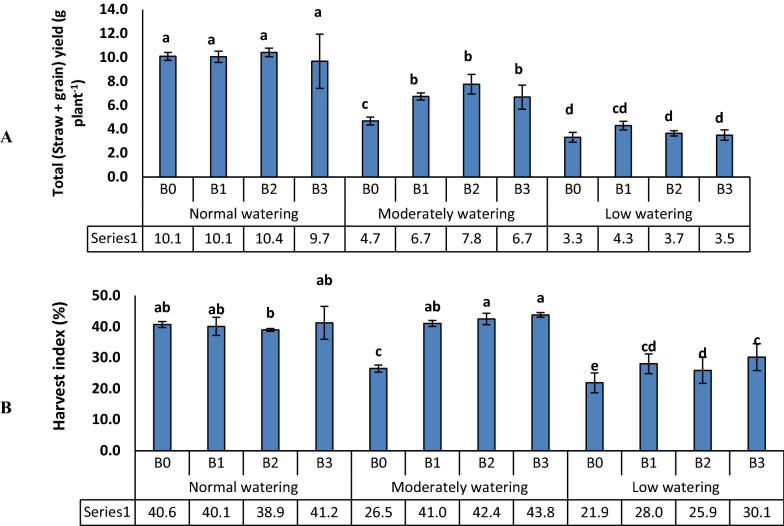
Dependence of straw and seed yield (**a**) and harvest index (**b**) on watering, wheat cultivar and rhizobacterial treatments. Wheat seedlings of the varieties Kohdasht (C1) and Chamran (C2) were grown at three levels of water availability and without (B0) or with the rhizobacteria strain 16-1 (B1), strain 38-2 (B2) and, strain 54-1 (B3). Different letters indicate a significant difference according to ANOVA and Duncan’s test (p < 0.05)

According to ANOVA, all measured root growth parameters, harvest index as well as straw and seed yield were affected by watering, and also by the bacteria except for root volume. By contrast, the wheat variety showed no effect on root tissue density or root water content (Table 3). Root growth parameters differed between the cultivars and watering levels, but the effects of bacteria were low. Root dry weight was lower in C1 than C2 and inhibited by water deficit (moderately and low watering) in both cultivars. With normal watering, root dry weight was decreased by B2 and B3 for C1 and by all bacteria for C2 compared to control. With moderate watering, root dry weight was increased by B3 in C1, but under low watering conditions it decreased with B1 inoculated C2 variety (Fig. 5a). Root volume was overall higher in C2 than C1, but was lower for both cultivars under water deficit compared to the control (Fig. 5b). Water deficit led to a decrease of root tissue density (Fig. 5c), as well as the inoculation of bacteria in high (80%) and low (30%) water availability in both wheat cultivars compared to the control in each water regimes. Finally, if compared to conditions of normal watering, root water content increased with water deficit in both cultivars (Fig. 5d). Bacterial influence was at its strongest at normal watering, when inoculation by all strains enhanced root water content. With moderately watering, the levels decreased by B1 and B3 in Kohdasht variety. By contrast with low watering, they increased by B1 in Kohdasht (C1), and by B2 in Chamran variety (C2). As expected, a decrease in total yield (straw + grain) occurred due to lower watering levels (Fig. 6a). Bacterial inoculation increased the yield at moderate drought, but not under severe drought or normal watering. By contrast, the positive influence of the bacteria on harvest index was evident in moderate and severe drought (Fig. 6b).

**Table 3:** Analysis of variance (ANOVA) of the yield and root parameters of wheat

Source of variation	df	Mean of square						
		Straw and grain yield	Harvest index	Root tissue density	Root volume	Root fresh weight	Root dry weight	Root water content
Watering	2	243.7**	1420**	0.004**	107.3**	44.9**	12.7**	0.56**
Bacteria	3	3.7**	178**	0.026**	3.6^ns^	2.9*	2.4**	0.43**
Wheat cv	1	387.8**	755**	0.001^ns^	304.5**	139.4**	26.7**	0.12^ns^
Watering × Bacteria	6	1.9*	64**	0.010**	2.6^ns^	1.7^ns^	1.4**	0.40**
Wheat cv. × Watering	2	36.8**	560**	0.020**	24.6**	4.2*	1.1**	0.39**
Bacteria × Wheat cv	3	4.2**	46**	0.002*	3.0^ns^	1.3^ns^	0.9**	0.14^ns^
Bacteria × Wheat cv. × Watering	6	0.5^ns^	43**	0.001^ns^	4.8*	1.7^ns^	0.4*	0.19**
CV (%)		13.7	9.4	10	20.2	20	20.5	14.9

Asterisks indicate significant differences according to ANOVA at the levels of p < 0.05* and p < 0.01**

### Localization of bacterial biofilms on wheat roots

**Fig. 7:**
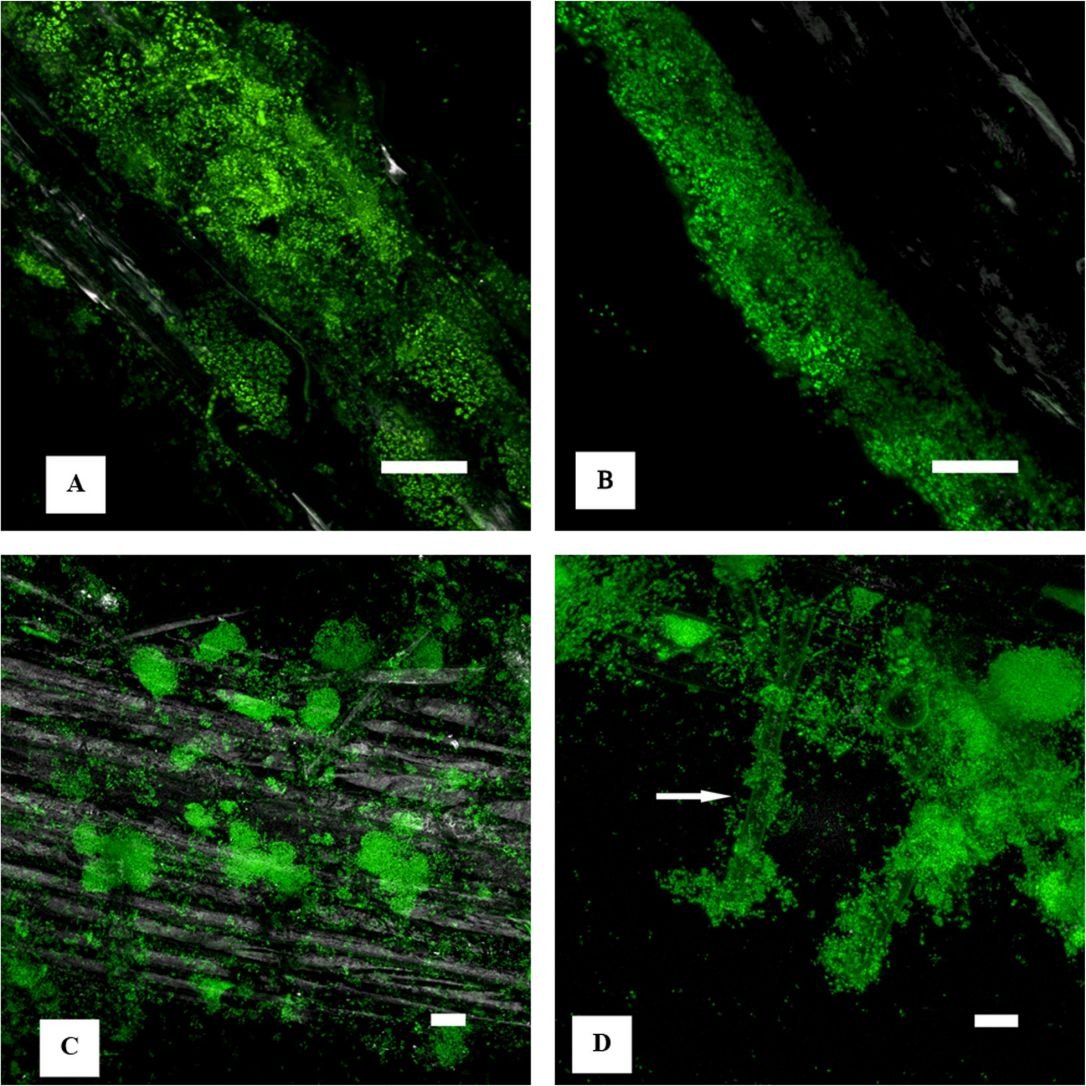
Confocal laser scanning microscopy of bacterial biofilm formation on wheat roots. Main roots colonized by strains 16-1 (B1: **A**, **B**) and 54-1 (B3: **C**, **D**) are shown as maximum intensity projection. Take notice of extended biofilms of B1 (**A**, **B**) and round/oval patterns indicating very dense microcolonies of B3 (**C**, **D**). Color allocation: nucleic acids—green, reflection—grey, arrow marks a root hair with associated bacteria, scale bar—20 µm

Two of the three promising candidates for wheat growth promotion, strain 16-1 (B1) and strain 54-1 (B3) were subjected to microscopical analysis. Their ability to root adherence and biofilm formation on wheat primary root were visualized by confocal laser scanning microscopy (Fig. 7). B1 (Fig. 7A, B) formed more extended biofilms covering larger areas of the wheat root, with no obvious pattern. In contrast, B3 (Fig. 7C, D) biofilms showed round or oval patterns, and compared to the strain B1 the bacterial cells were in most biofilm micro-colonies densely packed. These results strongly suggest that the PGPR strains of the current study form biofilms on wheat roots.

## Discussion

### Arid grassland rhizospheres support biofilm forming bacterial consortia

From the biofilm forming ability assay of the isolated bacteria it could be concluded that biofilm forming bacteria were abundant in the rhizospheres of grasses from an arid grassland. Our data thus adds to the literature that suggests the importance of biofilm formation for drought tolerance of rhizosphere bacteria. For instance, Chang et al. (2007) reported that hydrated microenvironment promotes EPS production by *Pseudomonas putida*, and contributes to biofilm architecture and stress tolerance under water limiting conditions. Their study also showed that EPS and alginate production increased with increasing matric potential. Roberson and Firestone (1992) showed that the soil *Pseudomonas* spp. from desiccated sand cultures produced more EPS than the ones from non-desiccated ones, and Schnider-Keel et al. (2001) confirmed that EPS production is central for the tolerance to desiccation and osmotic stress in *Pseudomonas fluorescens*. Khan and Bano (2019) showed that inoculation of two EPS producing PGPRs, *Planomicrobium chinense* strain P1 and *Bacillus cereus* strain P2, improves the physiological traits of wheat during drought stress, enhancing nutrient accumulation, relative water content, as well as leaf chlorophyll, protein and sugar contents.

### PGP traits of rhizospheric PGPR support growth of the host plant

PGP trait assesments of the isolated bacteria showed their strong potential in auxin production, ACC-deaminase activity, phosphorus solubilization, and K releasing from the unsoluble sources. Bacterial production of indole-3-acetic acid modulates root elongation and induces lateral root formation promoting root proliferation and altering root architecture (Sukumar et al. 2013). ACC deaminase inhibits ethylene production (Glick 2014). In contrast, enhanced ethylene production inhibits root elongation and leads to increased production of root hairs (Tanimoto et al. 1995). Thus, bacteria with ACC deaminase activity may promote root elongation, supporting the proliferation of the root system. ACC deaminase activity also suppresses stress ethylene levels, and the beneficial impacts of ACC deaminase active PGPR is often pronounced under environmental stress (Glick 2014; Yoolong et al. 2019). Danish and Zafar (2019) showed that co-application of two PGPR strains, *Agrobacterium fabrum* and *Bacillus amyloliquefaciens* with ACC deaminase activity increased chlorophyll a and b levels, photosynthetic rate, 1000-grain weight, as well as grain N, P and K content of wheat under drought conditions.

### Improving the drought resistance responsible physiological characteristics of wheat by biofilm forming bacterial inoculation

The results clearly showed that bacteria altered the physiological behavior of plants in normal as well as under water stress conditions. A decline in RWC as a criteria of plant water status in leaves reflects a loss of turgor that results in limited cell expansion and, consequentially, reduced growth of plants (Ma et al. 2012). Plants that are better adapted to drought have higher RWCs (Jarvis and Jarvis 1963). Some studies under water deficit proved that PGPR strains that improve survival of plants under water stress generally increase RWC in the plants. Grover et al. (2010) reported that sorghum plants, inoculated with *Bacillus* spp. strain KB 129, resulted in a 24% increase in RWC over non-inoculated plants under water deficit. Similar results have been demonstrated for maize (Sandhya et al. 2010), sunflower (Castillo et al. 2013), and wheat (Ilyas et al. 2020). Su et al. (2017) showed that inoculation of ryegrass by *B. amyloliquefaciens* kept the RWC at a high level even after 20 days of water deficit. Mechanisms behind an increase of RWC by bacteria include induced stomatal closure as a result of bacterial absicisic acid production (Dodd et al. 2010), the accumulation of cholin and glycine betaine (Gou et al. 2015) and enhanced water uptake by changes in the root traits (Glick 2014). Akhtar et al. (2020) showed that *Bacillus licheniformis* FMCH001 could improve water use efficieny of maize at well-watered as well as at drought stress conditions and consequently incresed the RWC content of leaves as well as yields of maize.

In line with our results, Wang et al. (2012) reported that a consortium of three PGPR strains on cucumber plants reduced the MDA content in leaves over control during water shortage. MDA content of wheat also decreased by the inoculation with *Dietzia natronolimnaea* STR1, both in normal and salt stress conditions (Bharti et al. 2016), and Su et al. (2017) reported that MSI of ryegrass leaves improved over 50% under severe drought stress upon inoculation with *B. amyloliquefaciens*. Water deficit leads to the generation of reactive oxygen species (ROS). In order to deal with the ROS, enzymatic and non-enzymatic components as antioxidant defense systems are activated (Miller et al. 2010). The effects of bacteria on the antioxidant enzyme activities but not all of them, were strongly modulated by plant cultivar and watering. An association of CAT production and drought tolerance has been observed in cucumber plants (Wang et al. 2012), maize (Sandhya et al. 2010), and wheat (Kasim et al. 2013). *D. natronolimnaea* STR1 enhanced CAT and APX activities of wheat plants under non-saline and saline conditions (Bharti et al. 2016). Furthermore, Ilyas et al. (2020) reported that combined wheat seed inoculation with two biofilm forming PGPR *Bacillus subtilis* (MT742976) and *Azospirillum brasilense* enhanced SOD activity by 35%, CAT by 77%, and POD by 40.7% vs. non-inoculated wheat, at 45% available water. Valente et al. (2019) have evaluated the interaction between the model PGPR strain, *Pseudomonas kilonensis* F113, with 199 ancient and modern wheat genotypes, and revealed that there is a particular high response by ancient wheat cultivars. Akbari et al. (2020) revealed specific effects of *Streptomyces rimosus* C-2012 on wheat cultivars. Although the strain C-2012 attenuated the negative effect of salt stress by stimulating APX and SOD activities as well as chlorophyll contents of both cultivars, it promoted the growth of only one cultivar. Similar results were also obtained by Egamberdieva (2010) with *Pseudomonas* sp. NUU1 and *Pseudomonas fluorescens* NUU2. Both isolates stimulated shoot and root length growth as well as increased dry weight of wheat cv. Turon, whereas cv. Residence was less affected by bacterial inoculation. In sum, we suggest that prior to a selection of bacterial inoculants for field applications, it is recommended to select responsive cultivars, those that benefit from the association with these bacteria.

Among the various plant growth-promoting bacteria, the members of the genus *Bacillus* have been among the most commonly commercialized. Their ability to form heat and desiccation-tolerant endospores distinguish them from the other groups of the PGPR. Various reports show how *Bacillus* spp. perform well under different environmental conditions (Xu and Cote 2003). For instance, Akinrinlola et al. (2018) reported that *Bacillus megaterium* R181, *B. safensis* R173*, Bacillus simplex* R180, and *Paenibacillus graminis* R200 increase the growth of wheat. These isolates enhanced shoot height, fresh weight of stem and roots. Wheat treated with EPS-producing bacterium *B. cereus* showed resistance to water stress through improvement in the physiological traits such ROS machinery, and reduced injuries of membrane lipids, and root system (Khan and Bano 2019). The underlying mechanisms are not only direct growth promotion by phosphate solubilization, siderophore, indole acetic acid, gibberellin and ACC deaminase production, but also indirect growth promotion by biocontrol based on to proteolytic and chitinolytic enzyme activities, hydrogen cyanide and biosurfactant production (Radhakrishnan et al. 2017).

### Root traits and yield of wheat plants responded to the bacterial inoculation

It has been reported that some of the PGPRs such as *B. pumilus* INR-7 are able to enhance lignin deposition (Zemrany et al. 2007). On the other hand, PGPRs can modify the chemical composition and therefore structural properties of plants root cell walls (Vacheron et al. 2013). Thus, it seems that inoculation of isolates to the plants in this study caused some structural variations in wheat cultivar’s root. Lozano et al. (2020) showed that as a response to water limitation, the grass root diameter increases and root tissue density decreases, i.e. a negative correlation between root tissue density and root diameter. Root tissue density is negatively related to soil fertility (Kramer-Walter et al. 2016), and lower tissue density may facilitate faster acquisition of nutrients, with the costs of shorter lifespan (Withington et al. 2006). In contrast, high root tissue density and large diameter were characteristic of plants exhibiting a long root lifespan and low growth rate (Fort et al. 2013). Thus, in the case of our study, the decrease in root tissue density by the *Bacillus* inoculation may improve the root’s ability for nutrient uptake under water deficit. Harvest index implies the relative distribution of photosynthesis products between economical sinks and the other existing sinks in the plant. Thus, the obtained data highlighted that the isolates could change the distribution of photosynthetic products in favor to grain, which led to an increase in the wheat grain yield. Some studies under water shortage stress have showed increasing in the shoot and plant growth by PGPRs inoculation with sorghum, sunflower, wheat and maize (Ngumbi and Kloepper 2016). Vardharajula et al. (2011) showed significantly greater shoot length and dry biomass under drought stress conditions with PGPRs compared to non-inoculated plants. Timmusk et al. (2014) showed that under drought stress, wheat plants treated with PGPR had 78% higher biomass if compared to non-treated plants. Lim and Kim (2013) highlighted that in pepper plants treated with *Bacillus licheniformis* K11 under water stress, shoot length was increased and had 50% more biomass than non-treated plants.

Based on the finding of our studies it would be concluded that rhizosphere microorganisms in arid ecosystems are commonly drought tolerant, rhizosphere compatible and have adapted to stress responses of their host plants. Consequently, applications with them represent an alternative and sustainable method to help plants cope with drought (de Vries et al. 2020). By isolating bacteria from grass rhizospheres in an arid ecosystem and filtering during the collection process for biofilm-forming bacteria with multiple plant growth promoting properties, we obtained three promising candidate strains for yield improvement of wheat. As expected, the positive influences of these bacteria were evident under moderate and/or severe water stress, suggesting that the hot and dry climate of East Azerbaijan grasslands promotes the development of drought tolerant PGPR. Since drought and high temperatures often coincide, future field work should show if the *Bacillus* strains promote wheat production at high temperatures and drought stress.

## Supplementary Information

**Additional file 1: Table S1. **The characteristics of the soil that was used in the greenhouse experiment.** Table S2.** Partial 16S sequences of the strains 16-1, 38-2 and 54-1. DSM numbers mark the accession numbers in the German Collection of Microorganisms and Cell Cultures, and B1–B3 indicate the terms used in plant inoculation experiments. **Table S3.** Bacterial ability (+) or inability (−) to grow on sorbitol. **Table S4.** Strongly adhering and biofilm-forming PGPR. Twenty-Four isolates with high biofilm forming activity and 40 Pellicle forming biofilm isolates. The isolates which were tested for their PGPR activity in a greenhouse experiment, are marked with bold letters. **Figure S1.** Sampling site of biofilm forming bacteria from Hashtroud county, East Azerbaijan province, northwestern Iran. **Figure S2. **Formation of pellicle biofilms in the test tube wall in TSB liquid culture medium. From right to left in two replications are the isolates 16-2, 38-1, and 42-1. Arrow indicate pellicle biofilms. **Figure S3. **Biofilms formed on the surface of the test tube. Purple color indicates the formation of biofilm. Left, isolate 54-1 and right, isolate 3-8. **Figure S4.** Morphological variation between the bacterial colonies in different concentrations of sorbitol. As an example, four pellicle forming bacteria (*Bacillus zhangzhouensis* 23-3, *Bacillus simplex* 22-1, *Paenibacillus lautus* 29-6 and *Bacillus simplex *55-1) are shown in the presence of 1, 10, 20 and 30 % sorbitol.

## Data Availability

The datasets generated during and/or analyzed during the current study are available from the corresponding author on reasonable request. The bacterial isolates 16-1, 38-2 and 54-1 are available at the German Collection of Microorganisms and Cell Cultures under the accessions DSM113966, DSM113967 and DSM113968, respectively.
