# Supplementary material for: Biofilm forming rhizobacteria affect the physiological and biochemical responses of wheat to drought
Source: AMB Express. 2022 Jul 14;12:93. doi: 10.1186/s13568-022-01432-8 (PMC9283637; doi:10.1186/s13568-022-01432-8)
Supplement: Supplementary file 1 — Additional file 1: Table S1. The characteristics of the soil that was used in the greenhouse experiment. Table S2. Partial 16S sequences of the strains 16-1, 38-2 and 54-1. DSM numbers mark the accession numbers in the German Collection of Microorganisms and Cell Cultures, and B1–B3 indicate the terms used in plant inoculation experiments. Table S3. Bacterial ability (+) or inability (−) to grow on sorbitol. Table S4. Strongly adhering and biofilm-forming PGPR. Twenty-Four isolates with high biofilm forming activity and 40 Pellicle forming biofilm isolates. The isolates which were tested for their PGPR activity in a greenhouse experiment, are marked with bold letters. Figure S1. Sampling site of biofilm forming bacteria from Hashtroud county, East Azerbaijan province, northwestern Iran. Figure S2. Formation of pellicle biofilms in the test tube wall in TSB liquid culture medium. From right to left in two replications are the isolates 16-2, 38-1, and 42-1. Arrow indicate pellicle biofilms. Figure S3. Biofilms formed on the surface of the test tube. Purple color indicates the formation of biofilm. Left, isolate 54-1 and right, isolate 3-8. Figure S4. Morphological variation between the bacterial colonies in different concentrations of sorbitol. As an example, four pellicle forming bacteria (Bacillus zhangzhouensis 23-3, Bacillus simplex 22-1, Paenibacillus lautus 29-6 and Bacillus simplex 55-1) are shown in the presence of 1, 10, 20 and 30 % sorbitol. [file 13568_2022_1432_MOESM1_ESM.docx]

**Journal name:**

AMB Express

**Manuscript Title:**

**Biofilm forming plant growth promoting rhizobacteria affect the physiological and biochemical responses, root traits and yield of wheat under drought**

**Authors:**

**Esmaeil Karimi^1^,**

**Nasser Aliasgharzad^2^,**

**Ezatollah Esfandiari^3^,**

**Mohammad Bagher Hassanpouraghdam^4^,**

**Thomas R. Neu^5^,**

**François Buscot^6,7^,**

**Thomas Reitz^6,7^,**

**Claudia Breitkreuz^6^, and**

**Mika T. Tarkka^6,7^**

^1^Department of Soil Science, University of Maragheh, Iran

^2^Department of Soil Science, University of Tabriz, Iran

^3^Department of Agronomy and Breeding, University of Maragheh, Iran

^4^Department of Horticultural Sciences, University of Maragheh, Iran

^5^Department of River Ecology, Helmholtz Centre for Environmental Research - UFZ, Magdeburg, Germany.

^6^Department of Soil Ecology, Helmholtz Centre for Environmental Research - UFZ, Halle, Germany

^7^German Centre for Integrative Biodiversity Research (iDiv) Halle-Jena-Leipzig, Leipzig, Germany

**Corresponding Author:**

Mika Tarkka, E-mail address: mika.tarkka@ufz.de, Tel.: +493455585414

**Table S1** The characteristics of the soil that was used in the greenhouse experiment.

| **Parameter** | **Value** |
| --- | --- |
| Sand (%) | 62 |
| Silt (%) | 21 |
| Clay (%) | 17 |
| Soil texture | Sandy Loam |
| θmFC (%) | 20 |
| OM (%) | 0.52 |
| pH | 7.54 |
| ECe (dS/m) | 0.55 |
| T.N.V (%) | 5 |
| P (mg. kg-1) | 16 |
| K (mg. kg-1) | 390 |
| Fe (mg. kg-1) | 4.92 |
| Zn (mg. kg-1) | 0.66 |
| Cu (mg. kg-1) | 0.94 |
| Mn (mg. kg-1) | 5.68 |

**Table S2** Partial 16S sequences of the strains 16-1, 38-2 and 54-1. DSM numbers mark the accession numbers in the German Collection of Microorganisms and Cell Cultures, and B1-B3 indicate the terms used in plant inoculation experiments.

>*Peribacillus simplex* strain 16-1 DSM113966 (B1)

AAAGTCTGACTGGAGCAACGCCGCGTGAACGAAGAAGGCCTTCGGGTCGTAAAGTTCTGT

TGTTAGGGAAGAACAAGTACCAGAGTAACTGCTGGTACCTTGACGGTACCTAACCAGAAA

GCCACGGCTAACTACGTGCCAGCAGCCGCGGTAATACGTAGGTGGCAAGCGTTGTCCGGA

ATTATTGGGCGTAAAGCGCGCGCAGGTGGTTCCTTAAGTCTGATGTGAAAGCCCACGGCT

CAACCGTGGAGGGTCATTGGAAACTGGGGAACTTGAGTGCAGAAGAGGAAAGTGGAATTC

CAAGTGTAGCGGTGAAATGCGTAGAGATTTGGAGGAACACCAGTGGCGAAGGCGACTTTC

TGGTCTGTAACTGACACTGAGGCGCGAAAGCGTGGGGAGCAAACAGGATTAGATACCCTG

GTAGTCCACGCCGTAAACGATGAGTGCTAAGTGTTAGAGGGTTTCCGCCCTTTAGTGCTG

CAGCTAACGCATTAAGCACTCCGCCTGGGGAGTACGGCCGCAAGGCTGAAACTCAAAGGA

ATTGACGGGGGCCCGCACAAGCGGTGGAGCATGTGGTTTAATTCGAAGCAACGCGAAGAA

CCTTACCAGGTCTTGACATCCTCTGACAACCCTAGAGATAGGGCTTTCCCCTTCGGGGGA

CAGAGTGACAGGTGGTGCATGGTTGTCGTCAGCTCGTGTCGTGAGATGTTGGGTTAAGTC

CCGCAACGAGCGCAACCCTTGATCTTAGTTGCCAGCATTCAGTTGGGCACTCTAAGGTGA

CTGCCGGTGACAAACCGGAGGAAGGTGGGGATGACGTCAAATCATCATGCCCCTTATGAC

CTGGGCTACACACGTGCTACAATGGATGGTACAAAGGGCTGCAAACCTGCGAAGGTAAGC

GAATCCCATAAAGCCATTCTCAGTTCGGATTGTAGGCTGCAACTCGCCTACATGAAGCCG

GAATCGCTAGTAATCGCGGATCAGCATGCCGCGGTGAATACGTTCCCGGGCCTTGTACAC

ACCGCCCGTCACACCACGAGAGTTTGTAACACCCGAAGTCGGTGAGGTAACCTTCATGGA

GCCAGCCGCCTAAGGTGG

>*Bacillus pumilus* strain 38-2 DSM113967 (B2)

AAGTCTGACGGAGCAACGCCGCGTGAGTGATGAAGGTTTTCGGATCGTAAAGCTCTGTTG

TTAGGGAAGAACAAGTGCGAGAGTAACTGCTCGCACCTTGACGGTACCTAACCAGAAAGC

CACGGCTAACTACGTGCCAGCAGCCGCGGTAATACGTAGGTGGCAAGCGTTGTCCGGAAT

TATTGGGCGTAAAGGGCTCGCAGGCGGTTTCTTAAGTCTGATGTGAAAGCCCCCGGCTCA

ACCGGGGAGGGTCATTGGAAACTGGGAAACTTGAGTGCAGAAGAGGAGAGTGGAATTCCA

CGTGTAGCGGTGAAATGCGTAGAGATGTGGAGGAACACCAGTGGCGAAGGCGACTCTCTG

GTCTGTAACTGACGCTGAGGAGCGAAAGCGTGGGGAGCGAACAGGATTAGATACCCTGGT

AGTCCACGCCGTAAACGATGAGTGCTAAGTGTTAGGGGGTTTCCGCCCCTTAGTGCTGCA

GCTAACGCATTAAGCACTCCGCCTGGGGAGTACGGTCGCAAGACTGAAACTCAAAGGAAT

TGACGGGGGCCCGCACAAGCGGTGGAGCATGTGGTTTAATTCGAAGCAACGCGAAGAACC

TTACCAGGTCTTGACATCCTCTGACAACCCTAGAGATAGGGCTTTCCCTTCGGGGACAGA

GTGACAGGTGGTGCATGGTTGTCGTCAGCTCGTGTCGTGAGATGTTGGGTTAAGTCCCGC

AACGAGCGCAACCCTTGATCTTAGTTGCCAGCATTTAGTTGGGCACTCTAAGGTGACTGC

CGGTGACAAACCGGAGGAAGGTGGGGATGACGTCAAATCATCATGCCCCTTATGACCTGG

GCTACACACGTGCTACAATGGACAGAACAAAGGGCTGCGAGACCGCAAGGTTTAGCCAAT

CCCATAAATCTGTTCTCAGTTCGGATCGCAGTCTGCAACTCGACTGCGTGAAGCTGGAAT

CGCTAGTAATCGCGGATCAGCATGCCGCGGTGAATACGTTCCCGGGCCTTGTACACACCG

CCCGTCACACCACGAGAGTTTGCAACACCCGAAGTCGGTGAGGTAACCTTTATGGAGCCG

GCCGCGAAGGGGGGCAGATGATTGG

>*Peribacillus simplex* strain 54-1 DSM113968 (B3)

AGTCTGACGGAGCAACGCCGCGTGAACGAAGAAGGCCTTCGGGTCGTAAAGTTCTGTTGT

TAGGGAAGAACAAGTACCAGAGTAACTGCTGGTACCTTGACGGTACCTAACCAGAAAGCC

ACGGCTAACTACGTGCCAGCAGCCGCGGTAATACGTAGGTGGCAAGCGTTGTCCGGAATT

ATTGGGCGTAAAGCGCGCGCAGGTGGTTCCTTAAGTCTGATGTGAAAGCCCACGGCTCAA

CCGTGGAGGGTCATTGGAAACTGGGGAACTTGAGTGCAGAAGAGGAAAGTGGAATTCCAA

GTGTAGCGGTGAAATGCGTAGAGATTTGGAGGAACACCAGTGGCGAAGGCGACTTTCTGG

TCTGTAACTGACACTGAGGCGCGAAAGCGTGGGGAGCAAACAGGATTAGATACCCTGGTA

GTCCACGCCGTAAACGATGAGTGCTAAGTGTTAGAGGGTTTCCGCCCTTTAGTGCTGCAG

CTAACGCATTAAGCACTCCGCCTGGGGAGTACGGCCGCAAGGCTGAAACTCAAAGGAATT

GACGGGGGCCCGCACAAGCGGTGGAGCATGTGGTTTAATTCGAAGCAACGCGAAGAACCT

TACCAGGTCTTGACATCCTCTGACAACCCTAGAGATAGGGCTTTCCCCTTCGGGGGACAG

AGTGACAGGTGGTGCATGGTTGTCGTCAGCTCGTGTCGTGAGATGTTGGGTTAAGTCCCG

CAACGAGCGCAACCCTTGATCTTAGTTGCCAGCATTCAGTTGGGCACTCTAAGGTGACTG

CCGGTGACAAACCGGAGGAAGGTGGGGATGACGTCAAATCATCATGCCCCTTATGACCTG

GGCTACACACGTGCTACAATGGATGGTACAAAGGGCTGCAAACCTGCGAAGGTAAGCGAA

TCCCATAAAGCCATTCTCAGTTCGGATTGCAGGCTGCAACTCGCCTGCATGAAGCCGGAA

TCGCTAGTAATCGCGGATCAGCATGCCGCGGTGAATACGTTCCCGGGCCTTGTACACACC

GCCCGTCACACCACGAGAGTTTGTAACACCCGAAGTCGGTGAGGTAACCTTCATGGAGCC

AGCCGCCTAAGGG

__________________________________________________________________________________________

**Table S3** Bacterial ability (+) or inability (-) to grow on sorbitol.

| **Strongly adherent biofilm forming bacteria** | | | | | **Pellicle biofilm forming bacteria** | | | |  |
| --- | --- | --- | --- | --- | --- | --- | --- | --- | --- |
| **Isolate** | **Sorbitol conc. (%)** | | | | **Isolate** | **Sorbitol conc. (%)** | | | |
|  | **1** | **10** | **20** | **30** |  | **1** | **10** | **20** | **30** |
| 3-1 | + | + | + | - | 2-2 | + | + | + | - |
| 6-2 | + | + | + | + | 3-3 | + | + | + | + |
| 10-1 | + | + | - | - | 3-4 | + | + | + | + |
| 15-3 | + | + | + | + | 4-1 | + | + | + | + |
| 16-2 | + | + | + | - | 6-1 | + | + | + | + |
| 24-2 | + | + | + | + | 8-3 | + | + | + | - |
| 27-1 | + | + | + | + | 10-2 | + | + | + | + |
| 29-2 | + | + | + | + | 17-5 | + | + | + | + |
| 29-4 | + | + | + | + | 18-1 | + | + | + | + |
| 30-1 | + | + | + | - | 18-2 | + | + | + | + |
| 32-1 | + | + | + | + | 19-2 | + | + | + | + |
| 33-1 | + | + | + | + | 20-2 | + | + | + | - |
| 34-2 | + | + | + | + | 22-1 | + | + | + | + |
| 38-2 | + | + | + | + | 22-2 | + | + | - | - |
| 40-1 | + | + | + | + | 23-3 | + | + | + | + |
| 48-1 | + | + | + | + | 24-1 | + | + | + | + |
| 48-2 | + | + | + | + | 25-1 | + | + | + | + |
| 49-1 | + | + | + | - | 25-3 | + | + | - | - |
| 49-2 | + | + | + | + | 29-3 | + | + | + | + |
| 50-1 | + | + | + | - | 29-6 | + | + | + | + |
| 52-2 | + | + | + | + | 31-2 | + | + | + | + |
| 54-1 | + | + | + | - | 32-3 | + | + | + | + |
| 55-1 | + | + | + | + | 34-2 | + | + | + | + |
| 56-1 | + | + | + | + | 34-3 | + | + | + | - |
|  |  |  |  |  | 36-2 | + | + | + | - |
|  |  |  |  |  | 39-2 | + | + | + | - |
|  |  |  |  |  | 42-2 | + | + | + | + |
|  |  |  |  |  | 42-3 | + | + | + | + |
|  |  |  |  |  | 43-1 | + | + | + | + |
|  |  |  |  |  | 44-2 | + | + | + | + |
|  |  |  |  |  | 45-2 | + | + | - | - |
|  |  |  |  |  | 45-4 | + | + | + | + |
|  |  |  |  |  | 47-1 | + | + | + | - |
|  |  |  |  |  | 47-2 | + | + | + | + |
|  |  |  |  |  | 48-2 | + | + | + | + |
|  |  |  |  |  | 50-2 | + | + | + | + |
|  |  |  |  |  | 52-1 | + | + | + | - |
|  |  |  |  |  | 53-1 | + | + | + | + |
|  |  |  |  |  | 53-2 | + | + | + | - |
|  |  |  |  |  | 56-2 | + | + | + | + |

**Table S4** Strongly adhering and biofilm-forming PGPR. Twenty-Four isolates with high biofilm forming activity and forty Pellicle forming biofilm isolates. The isolates which were tested for their PGPR activity in a greenhouse experiment, are marked with bold letters.

| **Isolate** | **Biofilm forming activity** | **Auxin production (mg/l)** | **Mineral P solubilization (mg/l )** | **ACC deaminase activity**  **(µmol/36 h)** | **K release from muscovite**  **(mg/l)** |
| --- | --- | --- | --- | --- | --- |
| 3-1 | 0.36 | 7.55 | 251.92 | 0.28 | 149.4 |
| 6-2 | 0.31 | 5.24 | 12.04 | 0.36 | 150.7 |
| 10-1 | 0.34 | 18.07 | 0 | 0.46 | 141.55 |
| 15-3 | 0.48 | 2.59 | 27.14 | 0.38 | 150.62 |
| **16-2** | **3.57** | **14.43** | **10.43** | **0.61** | **139.62** |
| 24-2 | 0.4 | 6.84 | 24.99 | 0.25 | 130.14 |
| 27-1 | 0.33 | 4.95 | 109.17 | 0.39 | 175.41 |
| 29-2 | 0.68 | 45.33 | 0 | 0.65 | 147.61 |
| 29-4 | 0.31 | 29.72 | 29.99 | 1.33 | 138.31 |
| 30-1 | 0.84 | 12.87 | 44.28 | 0.3 | 143.79 |
| 32-1 | 0.66 | 62.08 | 110.19 | 0.2 | 125.54 |
| 33-1 | 0.68 | 4.76 | 6.83 | 0.36 | 134.75 |
| 34-2 | 0.63 | 14.81 | 14.08 | 0.38 | 84.75 |
| **38-2** | **0.33** | **37.06** | **191.31** | **2.7** | **148.41** |
| 40-1 | 0.36 | 39.2 | 22.55 | 0.72 | 145.34 |
| 48-1 | 1.29 | 5.42 | 52.75 | 0.58 | 148.31 |
| 48-2 | 0.49 | 11.18 | 12.24 | 0.2 | 155.04 |
| 49-1 | 0.43 | 3.21 | 185.8 | 0.17 | 59.17 |
| 49-2 | 0.34 | 5.42 | 42.95 | 0.37 | 0 |
| 50-1 | 0.49 | 10.05 | 12.55 | 0.44 | 147.12 |
| 52-2 | 0.36 | 0.57 | 16.32 | 0.18 | 0 |
| **54-1** | **0.55** | **30.35** | **0** | **0.9** | **141.38** |
| 55-1 | 1.67 | 4.95 | 14.78 | 0.72 | 162.66 |
| 56-1 | 0.52 | 3.16 | 1.22 | 0.19 | 133.46 |

| **Isolate** | **Auxin production (mg/l)** | **Mineral P solubilization (mg/l )** | **ACC deaminase activity**  **(µmol/36 h)** | **K release from muscovite**  **(mg/l)** |
| --- | --- | --- | --- | --- |
| 2-2 | 14.15 | 5.43 | 0.60 | 146.5 |
| 3-3 | 15.16 | 21.20 | 0.57 | 128.56 |
| 3-4 | 17.95 | 13.48 | 0.00 | 129.85 |
| 4-1 | 15.42 | 11.20 | 0.49 | 117.11 |
| 6-1 | 17.82 | 13.70 | 1.47 | 148.30 |
| 8-3 | 19.25 | 11.09 | 1.25 | 111.5 |
| 10-2 | 14.10 | 16.96 | 0.00 | 108.5 |
| 17-5 | 13.51 | 16.09 | 1.21 | 112.77 |
| 18-1 | 16.94 | 17.61 | 0.41 | 151.76 |
| 18-2 | 17.13 | 10.22 | 0.83 | 103.25 |
| 19-2 | 15.05 | 5.87 | 0.83 | 170.92 |
| 20-2 | 15.65 | 35.80 | 0.46 | 139.16 |
| 22-1 | 10.12 | 13.48 | 0.22 | 155.02 |
| 22-2 | 19.59 | 18.91 | 0.36 | 112.07 |
| 23-3 | 20.14 | 14.46 | 0.95 | 150.71 |
| 24-1 | 14.46 | 32.24 | 0.56 | 114.46 |
| 25-1 | 18.63 | 11.30 | 1.25 | 130.14 |
| 25-3 | 13.67 | 19.18 | 1.56 | 119.61 |
| 29-3 | 15.47 | 16.96 | 0.00 | 125.09 |
| 29-6 | 17.17 | 16.52 | 1.94 | 93.28 |
| 31-2 | 32.19 | 153.26 | 0.79 | 139.27 |
| 32-3 | 14.78 | 12.93 | 0.50 | 65.98 |
| 34-2 | 14.86 | 1.09 | 0.00 | 0.04 |
| 34-3 | 22.79 | 0.00 | 0.56 | 134.75 |
| 36-2 | 13.98 | 17.61 | 1.24 | 133.8 |
| 39-2 | 23.98 | 14.57 | 2.14 | 125.41 |
| 42-2 | 21.76 | 18.91 | 0.43 | 136.99 |
| 42-3 | 15.42 | 10.22 | 0.90 | 147.56 |
| 43-1 | 11.20 | 28.97 | 1.35 | 0.00 |
| 44-2 | 15.19 | 37.61 | 0.67 | 121.52 |
| 45-2 | 9.94 | 21.85 | 0.44 | 117.64 |
| 45-4 | 19.72 | 26.30 | 1.04 | 130.60 |
| 47-1 | 9.70 | 6.30 | 0.57 | 0.00 |
| 47-2 | 22.95 | 2.83 | 0.82 | 121.10 |
| 48-2 | 20.14 | 32.17 | 0.73 | 130.60 |
| 50-2 | 17.22 | 13.04 | 1.66 | 137.17 |
| 52-1 | 39.60 | 5.43 | 0.54 | 150.77 |
| 53-1 | 10.73 | 20.76 | 0.80 | 124.04 |
| 53-2 | 17.23 | 6.52 | 0.59 | 127.64 |
| 56-2 | 10.32 | 8.04 | 0.99 | 140.10 |

**Table S4** Strongly adhering and biofilm-forming PGPR (continued).

*
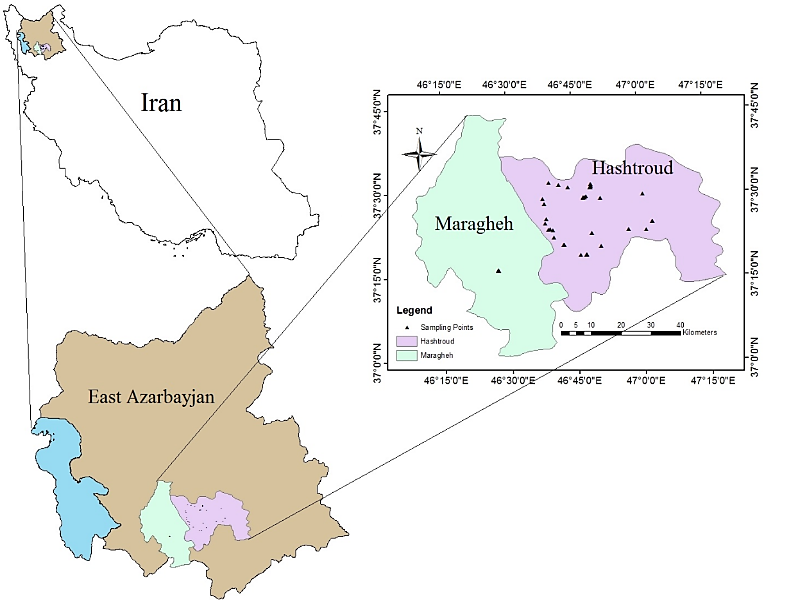
*

**Figure S1** Sampling site of biofilm forming bacteria from Hashtroud county, East Azerbaijan province, northwestern Iran.

*
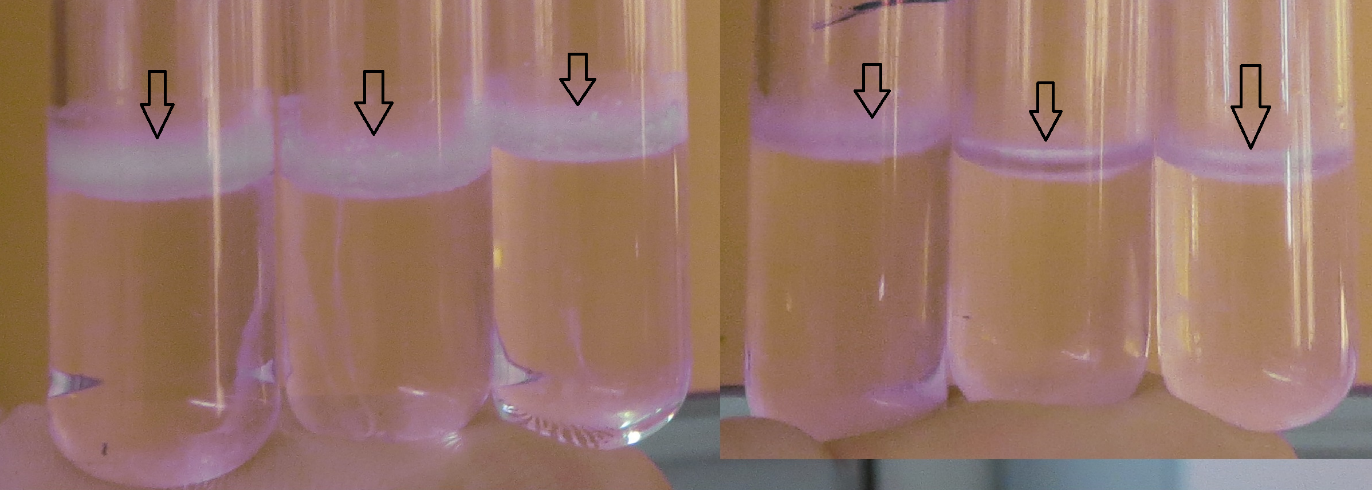
*

**Figure S2** Formation of pellicle biofilms in the test tube wall in TSB liquid culture medium. From right to left in two replications are the isolates 16-2, 38-1, and 42-1. Arrow indicate pellicle biofilms.

*
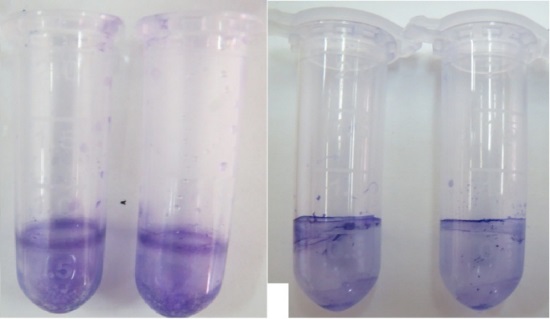
*

**Figure S3** Biofilms formed on the surface of the test tube. Purple color indicates the formation of biofilm. Left, isolate 54-1 and right, isolate 3-8

*
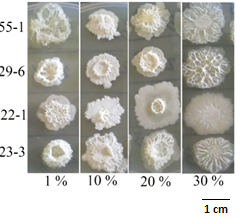
*

**Figure S4** Morphological variation between the bacterial colonies in different concentrations of sorbitol. As an example, four pellicle forming bacteria (*Bacillus zhangzhouensis* 23-3, *Bacillus simplex* 22-1, *Paenibacillus lautus* 29-6 and *Bacillus simplex* 55-1) are shown in the presence of 1, 10, 20 and 30 % sorbitol.
